# Evaluation of Palm Oil as a Suitable Vegetable Oil for Vitamin A Fortification Programs

**DOI:** 10.3390/nu8060378

**Published:** 2016-06-21

**Authors:** Marc Pignitter, Natalie Hernler, Mathias Zaunschirm, Julia Kienesberger, Mark Manuel Somoza, Klaus Kraemer, Veronika Somoza

**Affiliations:** 1 Department of Nutritional and Physiological Chemistry, Faculty of Chemistry, University of Vienna, Vienna 1090, Austria; hernler.natalie@gmail.com (N.H.); mathias.zaunschirm@univie.ac.at (M.Z.); Julia.Kienesberger@gmx.at (J.K.); veronika.somoza@univie.ac.at (V.S.); 2 Department of Inorganic Chemistry, Faculty of Chemistry, University of Vienna, Vienna 1090, Austria; mark.somoza@univie.ac.at; 3 Johns Hopkins Bloomberg School of Public Health, Baltimore, MD 21205, USA; klaus.kraemer@sightandlife.org; 4 Sight and Life, Kaiseraugst 4303, Switzerland

**Keywords:** palm oil, lipid oxidation, vitamin A, vitamin E, hexanal

## Abstract

Fortification programs are considered to be an effective strategy to mitigate vitamin A deficiency in populations at risk. Fortified vegetable oils rich in polyunsaturated fatty acids were shown to be prone to oxidation, leading to limited vitamin A stability. Thus, it was hypothesized that fortified oils consisting of mainly saturated fatty acids might enhance the stability of vitamin A. Mildly (peroxide value: 1.0 meq O_2_/kg) and highly (peroxide value: 7.5 meq O_2_/kg) oxidized palm oil was stored, after fortification with 60 International Units/g retinyl palmitate, in 0.5 L transparent polyethylene terephthalate bottles under cold fluorescent lighting (12 h/day) at 32 °C for 57 days. An increase of the peroxide value by 15 meq O_2_/kg, which was also reflected by a decrease of α-tocopherol congener by 15%–18%, was determined independent of the initial rancidity. The oxidative deterioration of the highly oxidized palm oil during storage was correlated with a significant 46% decline of the vitamin A content. However, household storage of mildly oxidized palm oil for two months did not induce any losses of vitamin A. Thus, mildly oxidized palm oil may be recommended for vitamin A fortification programs, when other sources of essential fatty acids are available.

## 1. Introduction

Vitamin A is involved in maintaining the function of epithelia, mucous membranes, immune and red blood cells and the visual system [1,2,3,4]. As vitamin A cannot be synthesized directly by the human body, it needs to be consumed in the form of animal source foods, such as eggs, liver and dairy products, or in the form of provitamin A from plant products rich in carotenoids, such as carrots, kale, spinach and sweet potatoes. Despite the poor bioavailability, bioconversion and bioefficacy of provitamin A, consumption of provitamin A rich plant foods alleviates vitamin A deficiency in low-income populations [5,6,7]. Vitamin A deficiency leads to severe health impairments, such as mortality and xerophthalmia [8,9]. The prevalence of vitamin A deficiency is generally associated with a lower socioeconomic status of a population. In south-east Asia, 45%–55% preschool children were affected by a severe vitamin A deficiency in 1995–2005 [8]. In Africa, a total of 2.55 million preschool-age children suffer from vitamin A deficiency-induced night blindness [8].

To combat vitamin A deficiency, the three main strategies, diet diversification (1), vitamin A supplementation (2) and food fortification (3), have been recommended, in addition to supporting other dietary approaches, such as home gardens and breastfeeding [10]. Dietary diversification (1) aims at educating populations at risk to consume sufficient amounts of micronutrients [11]. While nutrition education programs are a long-term approach, vitamin A supplementation (2) is considered a very effective short-term strategy [12]. Consumption of biofortified staple food crops (3) has been reported to improve the vitamin A status in children and women [13]. Fortification of staple foods with vitamin A is also a well-accepted and promising strategy to reduce vitamin A malnutrition [14]. Sugar, cereal-based products, margarine and edible oils have been commonly fortified with vitamin A [15,16,17]. To combat vitamin A malnutrition, vegetable oils are a good matrix, as vitamin A is fat-soluble and the oil can easily be fortified at low cost. Recently, fortification of vegetable oils with vitamin A has been suggested as an intervention strategy to increase the intake of vitamin A worldwide [18]; however, previous studies have demonstrated limited stability of vitamin A in soybean oil [19,20]. Laillou and colleagues [19] showed that storage of a mixture (1:1) of fortified sunflower and soybean oil characterized by a peroxide value (POV) of 0.4 meq O_2_/kg and exposed to sunlight for one month resulted in a 19.7% loss of vitamin A. The degradation of vitamin A was further enhanced, to 31.1%, when the same oil mixture, but with a higher POV of 5.8 meq O_2_/kg, was used. Another study reported a remarkable reduction of vitamin A of approximately 80% in soybean oil stored under household-representative conditions for 56 days [20]. While the daily decrease of retinol was about 1.5% in mildly oxidized oil, it was almost doubled when highly oxidized soybean oil was fortified with retinyl palmitate (RP).

The prominent loss of vitamin A during household storage of fortified vegetable oils prompted us to reconsider the appropriateness of vegetable oils rich in polyunsaturated fatty acids as food vehicle for vitamin A fortification. We hypothesized that edible oils that contain lower amounts of polyunsaturated fatty acids compared to soybean or sunflower oil might be less prone to oxidation, leading to an increased stability of vitamin A. Palm oil consists mainly of equivalent amounts of palmitic and oleic acids. The objective of the current study was to investigate the stability of RP in fortified palm oil stored under household conditions for 57 days. Mildly (POV < 2 meq O_2_/kg) and highly (POV > 5 meq O_2_/kg) oxidized palm oil was used to evaluate the impact of the oxidative status of the oil at the time of fortification on the stability of vitamin A. While storage of highly oxidized oil fortified with RP led to a significant decline of vitamin A, storage of mildly oxidized oil fortified with RP did not induce any significant losses of vitamin A.

## 2. Materials and Methods

### 2.1. Study Oil, Chemicals and Materials

Deodorized palm oil isolated from *Elaeis guineensis* was purchased from Natural Products & Drugs, Spittal/Drau, Austria. Fully deuterated hexanal (98 atom% D) was obtained from C/D/N Isotopes Inc., Quebec, Canada. All other chemicals were bought from Carl Roth, Karlsruhe, Germany, and Sigma-Aldrich, Vienna, Austria. The oil samples were illuminated using Philips cold fluorescent lamps (Philips, Amsterdam, Netherlands) with the following specifications: Tornado 1450 lumen, 103 W, spectrum from 400 to 650 nm. The oil was stored in 0.5 L transparent polyethylene terephthalate (PET) bottles (Radlberger, Unterradlberg, Austria). The bottles were closed with screw caps. The storage temperature was achieved with a radiator (AEG RA 5588, Electrolux Austria GmbH, Brunn am Gebirge, Austria) equipped with a temperature sensor to maintain the set temperature to the nearest of ±1.0 °C.

### 2.2. Study Design

**Figure 1:**
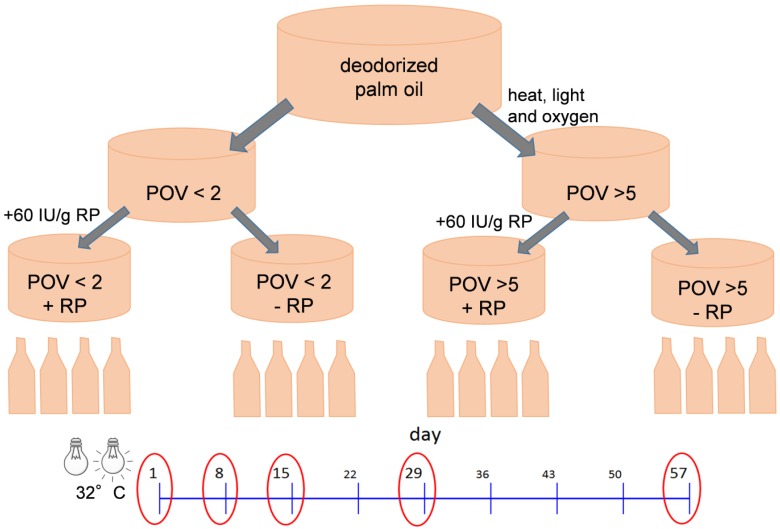
Study design. Four bottles filled with mildly (peroxide value (POV) < 2 meq O_2_/kg) or highly (POV > 5 meq O_2_/kg) oxidized palm oil, either fortified or not with 60 International Units (IU)/kg retinyl palmitate (RP), were stored under household-representative conditions under cold fluorescent light for 12 h per day at 32 °C for 57 days. Samples (40 mL) were withdrawn on a weekly basis, while samples from day 1, 8, 15, 29, and 57 were used for analyses.

To evaluate the stability of RP in mildly (POV 1.0 meq O_2_/kg) and highly oxidized (POV 7.5 meq O_2_/kg) palm oil, and the impact of RP fortification on the oxidative quality of the study oil, the mildly and highly oxidized palm oil were stored in the absence and presence of RP at 32 ± 2 °C for 57 days (Figure 1). The highly oxidized palm oil was obtained by vigorously stirring the oil under oxygen at approximately 150 °C for 4 h. The study oil was fortified with RP (60 International Units (IU)/g, 32.76 µg/mL) by preparing a premix, which was subsequently blended stepwise with palm oil, to obtain a homogeneous distribution of RP in the oil. Each bottle was filled with 447.5 ± 0.5 g of oil. During storage, the oil was exposed to cold fluorescent light for 12 h daily. The bottles were placed next to each other with a distance between each other of 15 cm. The distance between the light source and the bottles was 29 cm. To mimic consumer storage, the bottles were kept closed except for weekly opening for sampling. Samples from day 1, 8, 15, 29 and 57 were used for the analyses. A sample volume of 40 mL was drawn each week, thereby increasing the headspace. The samples were stored at −80 °C until analyses. Prior to analyses, the samples re-liquefied by gently heating at approximately 55 °C.

### 2.3. Determination of the POV

The POV was determined to study the effects of storage on the formation of primary lipid oxidation products in palm oil. The titrimetric measurement of the POV was performed as described previously [21,22]. Briefly, a saturated solution of potassium iodide was oxidized by 5 g of oil sample to yield iodine, which was titrated with a 0.1 N sodium thiosulfate solution in the presence of starch as an indicator. The POV was calculated by considering the amount of oil used and the concentration and the blank-corrected volume of sodium thiosulfate needed for discoloration of the solution. The POV was expressed as meq O_2_/kg. Aliquots of frozen oil samples were used as quality control.

### 2.4. Quantitation of the Fatty Acid Composition

The fatty acid composition was quantitated to characterize the palm oil, as well as to study the potential effect of storage of fortified and non-fortified palm oil on its fatty acid profile. The measurement of fatty acids was conducted as reported previously [22]. Briefly, the oil samples (100 mg) were spiked with 5 mg methylheptadecanoate and subjected to a base-catalyzed methylation before fatty acid methyl esters were extracted. The fatty acid methyl esters were analyzed by gas chromatography/flame ionization detection system (GC-2010 Plus, Shimadzu, Vienna, Austria) using a ZB-Wax Zebron capillary column (30 m × 0.25 mm × 0.25 µm, Phenomenex, Aschaffenburg, Germany). Quantitation of the fatty acid methyl esters were done by internal calibration. The recovery was determined to be 97.9% ± 2.89%. The limit of detection (LOD) and limit of quantitation (LOQ) values were determined according to the German Institute for Standardization (DIN 32645) and are listed in Table 1. Aliquots of frozen oil samples were used as quality control samples.

### 2.5. Quantitation of Hexanal

Hexanal was quantitated to investigate the impact of storage of non-fortified and fortified palm oil on the late stage of lipid oxidation. Measurement of hexanal was performed as described recently [22]. Briefly, the oil samples (2.5 g) were analyzed by static headspace gas chromatography-mass spectrometry (GCMS-QP 2010 Ultra, Shimadzu, Vienna, Austria) using the same column as for the fatty acid analyses. Quantitation of hexanal was performed applying stable isotope dilution analysis using hexanal-d12. The characteristic fragment ions of hexanal, *m/z* 72 and 56, and of hexanal-d12, *m/z* 80 and 64, were used for identification while, for quantitative purposes, *m/z* 72 and 80 were used for unlabeled and labeled hexanal, respectively. The response factor was calculated to be 1.45 ± 0.75. The recovery was determined to be 77.6% ± 17.9%. The LOD and LOQ values were determined according to the DIN 32645 and were 0.07 and 0.23 µg/mL, respectively. Aliquots of frozen oil samples were used as quality control samples.

### 2.6. Quantitation of Vitamin E

Vitamin E content was determined to study the impact of storage and retinol addition on its stability. Quantitation of vitamin E in palm oil was performed with slight modifications as described recently [22]. A sample amount of 0.05 g was dissolved in 1 mL isopropanol and analyzed by ultra high performance liquid chromatography (Dionex Ultimate 3000, Thermo Fisher Scientific, Vienna, Austria) at 295 nm using a C18 column (Kinetex EVO, 150 mm × 4.6 mm; 5 µm particle diameter, Phenomenex, Aschaffenburg, Germany). Quantitation of the tocopherol congeners were performed by external calibration. The recovery was determined to be 105% and 106% for α-tocopherol and γ-tocopherol, respectively. The LOD and LOQ values for α- and γ-tocopherol were determined according to the DIN 32645 and amounted 0.08 and 0.27 mg/kg for α-tocopherol, and 0.06 and 0.18 mg/kg for γ-tocopherol. In the oil samples, the δ-tocopherol concentration was below the LOD. Total tocopherols represent the sum of all detectable homologs. Aliquots of frozen oil samples were used as quality control samples.

### 2.7. Quantitation and Decomposition of Vitamin A

To investigate the stability of vitamin A palmitate in fortified palm oil, the oil samples were hydrolyzed prior to quantitation of retinol. Sample preparation for analysis of vitamin A were done as described elsewhere [23]. Briefly, 1 g oil sample was hydrolyzed at approximately 160 °C under reflux and nitrogen atmosphere for 30 min. The hydrolysate was extracted with hexane and dried before being diluted with methanol. The samples were analyzed with a triple quadrupole liquid chromatography-mass spectrometry system (LCMS-8040, Shimadzu, Vienna, Austria) using a C18 column (Kinetex EVO, 150 mm × 4.6 mm; 5 µm particle diameter, Phenomenex, Aschaffenburg, Germany). Retinol eluted at 7 min and was identified by applying multiple reaction monitoring transitions *m/z* 269.1 to 144.9 and *m/z* 269.1 to 133.0, while the latter transition was used for quantitation of retinol. External calibration was used for quantitation of retinol. The recovery was determined to be 72.3% ± 17.6%. The LOD and LOQ values were determined according to the DIN 32645 and amounted to 2.54 and 8.46 µg/mL, respectively. Mass scans in the range of *m/z* 100 to 2000 were used to determine the pseudo molecular ion of one of the main decomposition products from retinyl palmitate in palm oil. Aliquots of frozen fortified oil samples were used as quality control samples.

### 2.8. Statistical Analyses

All experiments were performed with four independent replicates. For the determination of fatty acids, tocopherols and vitamin A, two technical replicates were used. The results were expressed as mean ± standard error of mean (SEM) or as mean ± standard deviation (SD). Outliers were detected and removed from further analyses according to the results of the Nalimov outlier test. To identify statistically significant differences between day 1 and 57, a two-sided, paired *t*-test was applied. The impact of the oxidative status on the stability of RP as well as the effect of RP on the oxidative status of the palm oil was evaluated by one-way analysis of variance followed by the Holm-Sidak *post hoc* test using SigmaPlot 11 (Systat Software Inc., Chicago, IL, USA). Pearson product correlation coefficient was calculated between retinol concentration and tocopherol content. A *p*-value ≤ 0.05 described a statistically significant difference or correlation.

## 3. Results

### 3.1. Oxidative Stability of Mildly and Highly Oxidized Palm Oil Fortified with RP as Determined by the Fatty Acid Profile

The palm oil was characterized by quantitation of the most abundant fatty acids. The assay precision was calculated by the coefficient of variation of the quality control (QC) samples ranging from 5.2% to 7.1%. Table 2 shows the major fatty acids of the study oil on day 1 and 57. The most abundant fatty acids were oleic acid and palmitic acid with a proportion of approximately 85%. No change in the fatty acid profile was observed after storage of palm oil under household-representative conditions for two months.

### 3.2. Oxidative Stability of Mildly and Highly Oxidized Palm Oil Fortified with RP as Determined by the POV

**Figure 2:**
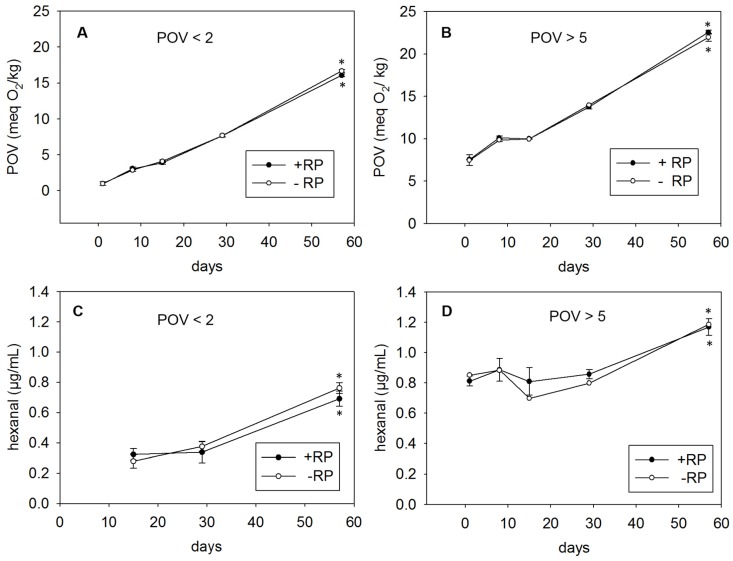
POV of mildly (**A**) and highly (**B**) oxidized palm oil and hexanal formation in mildly (**C**) and highly (**D**) oxidized palm oil in the presence or absence of RP after household-representative storage for up to 57 days. Data are expressed as mean ± standard deviation (SD) (*n* = 4). Asterisks (*) indicate a statistically significant difference *vs.* day 1 (*p* < 0.05).

The assay precision was calculated by the coefficient of variation of the QC samples ranging from 5.1% to 6.2%. Figure 2A shows a distinct increase of the POV in the palm oil after storage at 32 ± 2 °C for up to 57 days. While at the beginning of the study (day 1), the POV was determined to be 1.0 meq O_2_/kg, it increased to approximately 16 meq O_2_/kg after 57 days of storage. Figure 2B shows the results obtained from the highly oxidized palm oil. The POV of the highly oxidized oil increased from 7.5 meq O_2_/kg to approximately 22 meq O_2_/kg after household storage for 57 days. Although the POV of the highly oxidized palm oil (22 meq O_2_/kg) was higher at day 57 compared to the mildly oxidized palm oil (16 meq O_2_/kg), the average increase of the POV after 57 days was similar for both study oils, amounting approximately to 15 meq O_2_/kg. The POV of neither the mildly nor the highly oxidized palm oil was affected by the addition of 60 IU/g RP (Figure 2A,B).

### 3.3. Oxidative Stability of Mildly and Highly Oxidized Palm Oil Fortified with RP as Determined by a Secondary Lipid Oxidation Marker

Figure 2C demonstrates a significant increase of the hexanal formation from approximately 0.3 µg/mL on day 14 to 0.7 µg/mL on day 57. To verify an acceptable assay precision, the coefficient of variation of the QC samples were calculated to range between 5.9% and 7.6%. On the first two sampling days, the hexanal concentration was below the LOD of 0.07 µg/mL. Even storage of highly oxidized palm oil under household conditions revealed a significant increase of the hexanal formation only after 57 days (Figure 2D). Fortification of the mildly or highly oxidized palm oil with RP did not lead to any changes of the storage-induced generation of hexanal. As for the primary lipid oxidation products, vitamin A did not exhibit any antioxidant activities on the linoleic acid-derived hexanal.

### 3.4. Oxidative Stability of Mildly and Highly Oxidized Palm Oil Fortified with RP as Determined by the Vitamin E Content

**Figure 3:**
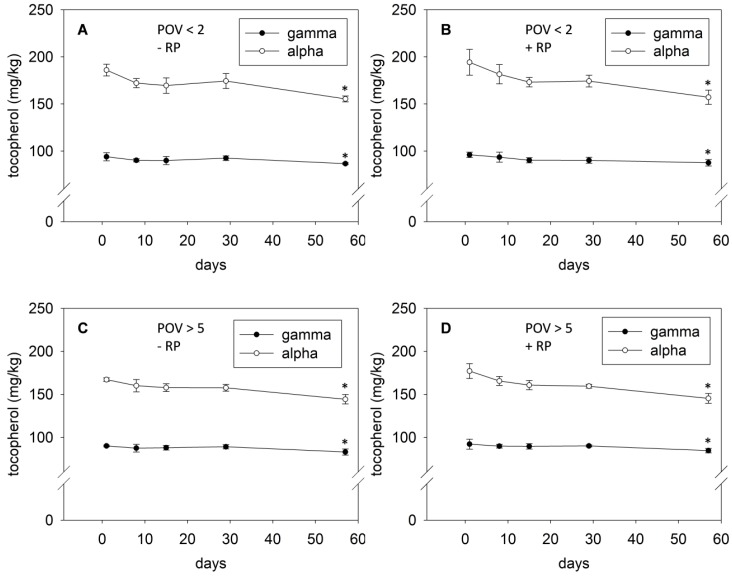
Decrease of α- and γ-tocopherol in mildly (**A**,**B**) and highly (**C**,**D**) oxidized palm oil either fortified (**B**,**D**) or not (**A**,**C**) with retinyl palmitate (RP) after household-representative storage for up to 57 days. Data are expressed as mean ± SD (*n* = 4). Asterisks (*) indicate a statistically significant difference *vs.* day 1 (*p* < 0.05).

For the vitamin E quantitation, the precision of the QC samples ranged from 8.0% to 10.4%. Figure 3A shows a slight decrease of the α-tocopherol concentration of approximately 18% in mildly oxidized palm oil stored under household-representative conditions for 57 days. The concentration of the other detectable tocopherol congener, γ-tocopherol, remained almost stable with a decline of only 8% after 57 days of storage.

In the present study, the marginal decrease of tocopherol congeners in palm oil during storage was not affected by the addition of RP on day 1, suggesting the absence of antioxidant activity of vitamin A (Figure 3B). Figure 3C depicts the effect of the oxidative status of the oil at day 1 on the stability of vitamin E. Similar to the results obtained by the two other lipid oxidation markers, the storage of the highly oxidized palm oil did cause a significant change in the vitamin E stability. Storage of the highly oxidized oil diminished the concentration of α-tocopherol by 15%, while the concentration of γ-tocopherol was reduced by 8%. A tocopherol-preserving effect of vitamin A, which was reported in soybean oil after household-representative storage [24], was hardly observed in highly oxidized palm oil during storage (Figure 3D).

**Figure 4:**
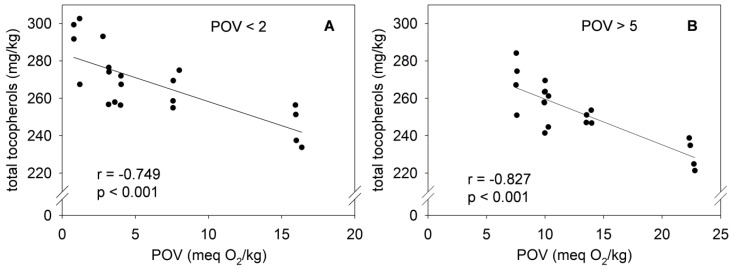
Correlation between the POV and the tocopherol content in mildly (**A**) and highly (**B**) oxidized palm oil stored under household-representative conditions in the presence of RP for 57 days.

The slight decline of the tocopherol homologs in mildly (Figure 4A) and highly (Figure 4B) oxidized palm oil was associated with an increase of the POV. The total tocopherol concentrations in the mildly oxidized palm oil stored for 57 days were negatively correlated with the POV (*r* = −0.749; *p* < 0.001). A correlation coefficient of −0.827 (*p* < 0.001) was also obtained between the total tocopherol concentrations and the POV in the highly oxidized palm oil.

### 3.5. Effect of the Oxidative Status of Palm Oil on the Stability of Vitamin A

**Figure 5:**
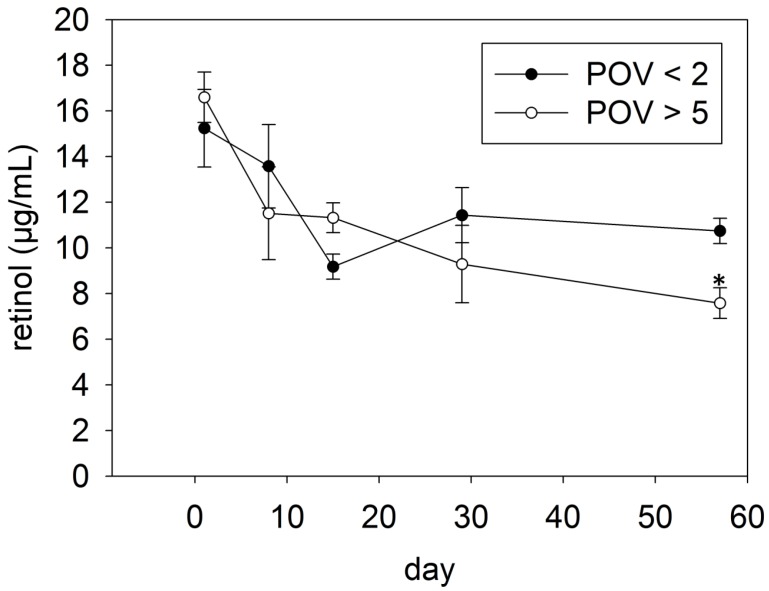
Vitamin A stability in mildly and highly oxidized palm oil stored under household-representative conditions for up to 57 days. Data are expressed as mean ± SEM (*n* = 4). Asterisks (*) indicate a statistically significant difference *vs.* day 1 (*p* < 0.05).

**Figure 6:**
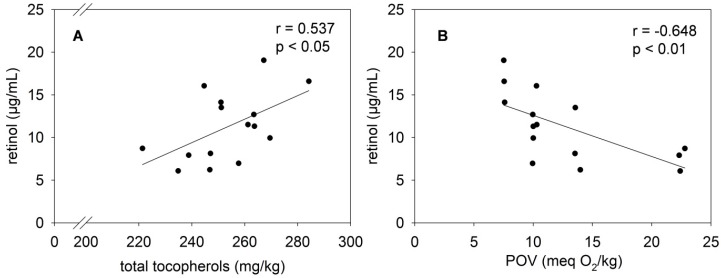
Correlation between the retinol concentration and the total tocopherol content (**A**) and the POV (**B**) in highly oxidized palm oil stored under household-representative conditions in the presence of RP for 57 days.

Figure 5 shows the stability of RP in mildly and highly oxidized fortified palm oil during storage for 57 days. The assay precision was calculated by the coefficient of variation of the QC samples ranging from 11.5% to 15.7%. The storage-induced vitamin A loss was inhibited when mildly oxidized palm oil was used for fortification. Although vitamin A tended (*p* = 0.095) to decrease during storage, the decline was not significant. However, using highly oxidized palm oil for fortification with RP resulted in a marked vitamin A loss, approximately 46%, after 57 days of storage. It could be shown that the loss of vitamin A during the storage of highly oxidized palm oil correlated with the tocopherol decay (Figure 6A; *r* = 0.537; *p* < 0.05), as well as with the rise of the POV (Figure 6B; *r* = −0.648; *p* < 0.01).

In the current study, we also provide first hints of the potential chemical identity of one of the degradation products of vitamin A in fortified, highly oxidized palm oil stored under household conditions for up to two months. Mass spectrometric analyses revealed the appearance of *m/z* 642 and 321, which were absent on day 1, in palm oil stored for eight days.

## 4. Discussion

The present study evaluated the suitability of palm oil as food vehicle for vitamin A fortification. Palm oil was chosen as vehicle as it contains a considerable amount of saturated fatty acids. Oleic acid and palmitic acid, the most abundant fatty acids in palm oil, are known to be less prone to oxidation compared to, e.g. linoleic and linolenic acid in soybean oil [25]. Thus, palm oil might be more resistant to oxidative rancidity than soybean oil, which consists of approximately 60% polyunsaturated fatty acids [22]. Nevertheless, the palm oil contained 111 mg/mL polyunsaturated fatty acids, requiring an intake of approximately 4 tablespoons of palm oil per day to achieve the recommended daily intake of 2 energy% linoleic acid [26]. As the fatty acids of the unfortified and fortified palm oil remained unchanged during storage, it might be conceivable that the oxidative quality of the palm oil is stable throughout the study. However, a recent study demonstrated that non-detectable changes in the fatty acid profile might not exclude oxidative deterioration of a vegetable oil [22]. This might be explained by the high sensitivity of the methods targeting lipid oxidation products [27]. Another reason might be the rather small proportion of fatty acids subjected to oxidation under household-representative storage condition compared to the total amount of fatty acids, leading to little or no detectable changes of the fatty acid profile but to a remarkable increase of lipid oxidation products.

To determine oxidative deterioration of the palm oil stored under household-representative conditions for 57 days, the POV was determined at the beginning, during and at the end of the present study. According to the Codex Alimentarius International Food Standards published by the World Health Organization and Food and Agriculture Organization of the United Nations, edible oils with a POV > 10 meq O_2_/kg are not recommended for dietary intake [28]. Therefore, based on the results from the current study, storage of palm oil under household conditions for more than one month may not be advisable. Another study on soybean oil identified cold fluorescent light as a more important factor in promoting lipid oxidation than high ambient temperature [22]. Thus, decreasing the storage temperature from 32 to 22 °C might not have led to an acceptable POV below 10 meq O_2_/kg in the present study. However, the oxidative stability of palm oil (POV of 16 meq O_2_/kg) was higher than that of soybean oil (POV of 28 meq O_2_/kg) stored under similar household conditions in the presence of cold fluorescent light at 32 °C for two months [20].

To investigate whether the progress of lipid oxidation of the edible oil might be affected by its initial degree of oxidative rancidity, the POV was also determined for the highly oxidized palm oil, characterized by a POV of 7.5 meq O_2_/kg. We hypothesized that enhanced oxidative deterioration of the vegetable oil might propagate and accelerate lipid oxidation due to the presence of lipid radicals. However, the different oxidative status of the two study oils on day 1 did not affect the progress of lipid oxidation as evident by an increase of the POV by 15 meq O_2_/kg after 57 days in both oils. In both study oils, the POV exceeded the acceptable threshold of 10 meq O_2_/kg after only one month of storage. With regard to the highly oxidized palm oil, this threshold was even reached after household-representative storage for eight days. According to our results, storage of mildly oxidized palm oil for more than one month is not advisable due to the formation of potentially harmful primary and secondary lipid oxidation products [29]. However, it can be assumed that consumers store edible oils for more than one month. Besides household storage, the storage in the supermarket needs to be considered as well. The expiry date does not take into account the formation of lipid oxidation products during household-representative storage, which is characterized by periodical openings of the bottle thereby facilitating oxidation. Although in the current study, the palm oil was purchased from the retail market, consumption of the oil after one month of storage within the expiration date could not be recommended. Consumption of oxidized lipids was associated with health-related implications [30]. For instance, feeding oxidized lipids was associated with a 100% increase in fatty streak lesions in the aorta of rabbits, thereby being an important risk factor for arteriosclerosis [31]. Results from these studies suggest that consumption of oxidized oil might promote cardiovascular diseases. Thus, it is of interest to alleviate lipid oxidation by addition of antioxidants.

Recently, an antioxidant activity of RP has been demonstrated by a significantly lower POV in fortified sunflower and soybean oil (6.18 ± 0.05 meq O_2_/kg) in comparison to unfortified oil (7.30 ± 0.10 meq O_2_/kg) after 30 days of accelerated storage in a Schaal oven at 60 °C [19]. We did not obtain any impact of the addition of RP to the study oils on the POV. The antioxidant activity of RP might only be detectable after the harsh storage conditions with elevated storage temperature as in the Schaal oven test [19].

To verify the effects of storage and vitamin A fortification on the oxidative stability of the mildly and highly oxidized oil, not only POV, as a marker for primary lipid oxidation, but also the formation of hexanal, which represents a marker for late stage lipid oxidation, was determined. Hexanal is a decomposition product of linoleic acid [32], which is one of the most prominent fatty acids in palm oil. As expected, the secondary lipid oxidation product hexanal only increased at a later stage of lipid oxidation. While in linoleic acid-rich soybean oil, the hexanal generation was detected during household-representative storage on day 1 and peaked on day 28 (approximately 3 µg/mL hexanal) [20], palm oil was less affected to oxidation as evident by the late rise of hexanal and the lower concentration detected compared to soybean oil. Thus, by comparing the results of the formation of the primary and secondary lipid oxidation marker of the present and previous study, a higher oxidative stability of palm oil compared to soybean oil could be confirmed, most likely due to the low amount of polyunsaturated fatty acids compared to soybean oil [20].

Vitamin E is also commonly considered as outcome measure of lipid oxidation in plant oils. The electron-donating activity of vitamin E allows it to scavenge free lipid radicals, thereby impeding propagation of lipid oxidation. Thus, vitamin E should protect the oil from oxidative deterioration due to its antioxidant activity. The high stability of tocopherols in palm oil, as evident from the current study, compared to the tocopherols in soybean oil, as demonstrated in one of our previous studies [22] might be explained by a higher proportion of saturated and monounsaturated fatty acids in palm oil, which makes it less prone to oxidation. As a consequence, the amount of lipid radicals generated in palm oil during storage might be limited, leading to a reduced chain-breaking activity of vitamin E. The higher degradation rate of α-tocopherol (0.32%/day) compared to γ-tocopherol (0.14%/day) in the present study is in agreement with others [33,34]. Player *et al.* [33] determined a degradation rate of 5.6 and 1.2%/day for α-tocopherol and γ-tocopherol, respectively, in soybean oil during storage at 50 °C for ten days. As the bond dissociation enthalpy of the hydroxyl group at the chromanol ring amounts to 76 and 78 kcal/mole for α-tocopherol and γ-tocopherol, respectively, the hydrogen-donating ability of α-tocopherol is higher compared to γ-tocopherol [35]. Therefore, α-tocopherol is preferably degraded in the presence of oxidizing compounds.

Due to the low oxidative rancidity of the fortified palm oil during storage under household-representative conditions for two months, we hypothesized that vitamin A might also show a high stability due to reduced exposure to lipid oxidation products compared to oils rich in unstable polyunsaturated fatty acids. In the present study, it was shown that the vitamin A stability in the palm oil studied depends on the progress of lipid oxidation as evident by the significant correlation between the lipid oxidation markers and the retinol concentration. The impact of the oxidative status of the edible oil on the vitamin A stability was also reported earlier [20,36]. Andarwulan and colleagues [37] observed a comparable loss of 43% of vitamin A in bulk, unbranded palm oils with initial oxidation levels of 0, 4 and 9 meq O_2_/kg after 53, 25 and less than 14 days of accelerated storage in the dark at 60 °C under nitrogen atmosphere, respectively. The current study focused not on forced oxidation conditions but rather on a household-representative storage environment at 32 °C, which might explain the absence of significant changes in the vitamin A content of mildly oxidized palm oil stored for up to 57 days. Hemery and colleagues [36] reported a 60%–68% loss of vitamin A in three fortified soybean oils, characterized by initial POV values of 1.63 ± 0.05, 2.75 ± 0.12 and 5.75 ± 0.04 meq O_2_/kg after storage in the dark for one month followed by another month of storage in the semi-dark or presence of sunlight. This discrepancy between the vitamin A stability in soybean oil rich in polyunsaturated fatty acids and in palm oil rich in saturated and monounsaturated fatty acids is also corroborated by one of our previous studies [20]. A vitamin A loss of more than 80% was reported after storage of fortified soybean oil under household conditions for 56 days [20].

In the current study, one of the retinol degradation products was detected by mass spectrometry. We hypothesize that *m/z* 642 and 321 might correspond to the dimeric retinol hydroperoxide and the monomeric radical, respectively, which might be predominantly formed in highly oxidized palm oil [38].

## 5. Conclusions

With regard to the nutritional value of vegetable oils, it is recommended to consume edible oils rich in polyunsaturated fatty acids. Thus, soybean oil should be preferably used as a vehicle for fortification with vitamin A. However, taking into account a successful delivery of vitamin A, vegetable oils rich in saturated fatty acids, such as palm oil, are preferred vehicles for the fortification with vitamin A compared to edible oils rich in polyunsaturated fatty acids. While household-representative storage of fortified soybean oil for two months led to a substantial loss of vitamin A, similar storage conditions of fortified palm oil did not induce statistically significant changes of the vitamin A concentration. Thus, we conclude that palm oil is superior to soybean oil for vitamin A fortification programs to deliver sufficient amounts of vitamin A. However, care needs to be taken not to store vegetable oils longer than one month under household conditions, independent of the degree of saturation of the fatty acids, as the peroxide value increases beyond the acceptable value of 10 meg O_2_/kg, and other dietary sources of essential fatty acids should be sought for to meet their needs.

## Figures and Tables

**Table 1:** Limit of detection and limit of quantitation values for the determination of methylated fatty acids in palm oil.

	LOD (µg/mL)	LOQ (µg/mL)
Methyl myristate	0.004	0.013
Methyl palmitate	0.072	0.240
Methyl palmitoleate	0.005	0.017
Methyl stearate	0.028	0.093
Methyl oleate	0.098	0.327
Methyl linoleate	0.013	0.043
Methyl linolenate	0.005	0.017

**Table 2:** Fatty acid composition in non-fortified palm oil with different initial oxidation levels.

Fatty Acid	Peroxide value < 2			Peroxide value > 5		
	Day 1 (mg/mL)	Day 57 (mg/mL)	Change (%)	Day 1 (mg/mL)	Day 57 (mg/mL)	Change (%)
						
C14:0	8.61 ± 0.41	8.34 ± 0.45	2.97 ± 5.04	8.56 ± 0.27	8.71 ± 0.40	−1.86 ± 5.69
						
C16:0	484 ± 25.0	464 ± 24.6	3.98 ± 5.75	479 ± 23.8	489 ± 23.3	−4.12 ± 6.25
						
C16:1	2.08 ± 0.08	2.05 ± 0.07	1.62 ± 3.74	2.08 ± 0.04	2.13 ± 0.09	−2.54 ± 4.85
						
C18:0	62.9 ± 3.25	60.5 ± 3.03	3.55 ± 5.54	62.2 ± 2.45	63.6 ± 2.93	−2.30 ± 6.13
						
C18:1	535 ± 26.6	514 ± 26.1	3.88 ± 5.77	529 ± 21.6	540 ± 25.9	−2.15 ± 6.32
						
C18:2	108 ± 5.20	102 ± 4.97	5.54 ± 5.27	105 ± 4.78	106 ± 5.06	−2.93 ± 5.88
						
C18:3	3.13 ± 0.10	2.86 ± 0.11	8.45 ± 6.22	2.93 ± 0.10	2.92 ± 0.13	0.20 ± 5.28

No statistically significant differences were detected. Results were expressed as mean ± standard deviation.

## Abbreviations

The following abbreviations are used in this manuscript:

DIN	German Institute of Standardization
LOD	limit of detection
LOQ	limit of quantitation
meq	milliequivalents
POV	peroxide value
RP	retinyl palmitate
